# Bt Jute Expressing Fused δ-Endotoxin Cry1Ab/Ac for Resistance to *Lepidopteran* Pests

**DOI:** 10.3389/fpls.2017.02188

**Published:** 2018-01-04

**Authors:** Shuvobrata Majumder, Chirabrata Sarkar, Prosanta Saha, Bheemanna S. Gotyal, Subrata Satpathy, Karabi Datta, Swapan K. Datta

**Affiliations:** ^1^Laboratory of Translational Research on Transgenic Crops, Department of Botany, University of Calcutta, Kolkata, India; ^2^Division of Crop Protection, Central Research Institute for Jute and Allied Fibres, Indian Council of Agricultural Research, Kolkata, India; ^3^Department of Crop Sciences, Institute of Agriculture, Visva Bharati University, Santiniketan, India

**Keywords:** Bt jute, *Spilarctia obliqua*, *Anomis sabulifera*, *Spodoptera exigua*, Bacillus thuringiensis, insect resistant plant, *cry1Ab/Ac*, integrated pest management

## Abstract

Jute (*Corchorus* sp.) is naturally occurring, biodegradable, lignocellulosic-long, silky, golden shiny fiber producing plant that has great demands globally. Paper and textile industries are interested in jute because of the easy availability, non-toxicity and high yield of cellulosic biomass produced per acre in cultivation. Jute is the major and most industrially used bast fiber-producing crop in the world and it needs protection from insect pest infestation that decreases its yield and quality. Single locus integration of the synthetically fused *cry1Ab/Ac* gene of *Bacillus thuringiensis* (Bt) in *Corchorus capsularis* (JRC 321) by *Agrobacterium tumefaciens-*mediated shoot tip transformation provided 5 potent Bt jute lines BT1, BT2, BT4, BT7 and BT8. These lines consistently expressed the Cry1Ab/Ac endotoxin ranging from 0.16 to 0.35 ng/mg of leaf, in the following generations (analyzed upto T_4_). The effect of Cry1Ab/Ac endotoxin was studied against 3 major *Lepidopteran* pests of jute- semilooper (*Anomis sabulifera* Guenee), hairy caterpillar (*Spilarctia obliqua* Walker) and indigo caterpillar (*Spodoptera exigua* Hubner) by detached leaf and whole plant insect bioassay on greenhouse-grown transgenic plants. Results confirm that larvae feeding on transgenic plants had lower food consumption, body size, body weight and dry weight of excreta compared to non-transgenic controls. Insect mortality range among transgenic feeders was 66–100% for semilooper and hairy caterpillar and 87.50% for indigo caterpillar. Apart from insect resistance, the transgenic plants were at par with control plants in terms of agronomic parameters and fiber quality. Hence, these Bt jutes in the field would survive *Lepidopteran* pest infestation, minimize harmful pesticide usage and yield good quality fiber.

## Introduction

Jute is the highest bast fiber-producing crop in the world. It is the second most important fiber crop after cotton. Jute fibers are naturally occurring, biodegradable, easily available and non-toxic. Among more than 170 species, only *Corchorus capsularis* (white jute) and *C. olitorius* (tossa jute) are cultivated in the major fiber producing countries. Jute fiber and its products have global demand in Australia, Germany, Japan, Russia, Spain, United Kingdom, and United States, because of the environment-friendly nature of the fibers. For the year 2013–2014, 99.82% of the total global jute production was from the developing countries of Asia, where India contributed 56.85% (1944 thousand tones), Bangladesh 40.67% (1391 thousand tones), China 1.03% (35.5 thousand tones), Uzbekistan 0.61% (21 thousand tones) and Nepal 0.45% (15.5 thousand tones) (FAO, 2017).

Jute cultivation faces the adverse effect of biotic and abiotic stresses. In recent years, due to the gradual shift in climatic parameters during the growing season and the radical change in input use pattern, jute crop has experienced changes in relative pest status of many insect pests (Satpathy et al., 2016). Further, some minor pests have elevated infestation and are now considered as major pests. The spectrum of pests that infest jute has increased with infestations from insects like the *Helicoverpa armigera* (Hubner) that has recently been reported (Selvaraj et al., 2013). Of all the jute *Lepidopteran* pests, semilooper (SL), hairy caterpillar (HC) and indigo caterpillar (IC) are the most potent destroyers at different stages of plant growth (Rahman and Khan, 2012; Selvaraj et al., 2015). The SL, being the most active foliage destroyer of jute, in its third instar stage, causes 48.5% fiber yield loss. It induces profuse plant branching (up to 90%) leading to breakage of fibers during extraction that causes decline in fiber quality (Tripathi and Ram, 1972). HC, a major polyphagous pest, causes up to 30% yield loss in jute cultivation (Bandyopadhyay et al., 2014). The third and fourth instar larvae of HC feed voraciously on leaves and spread rapidly to the entire plantation. Both the cultivated species of jute are highly susceptible to HC (Selvaraj et al., 2015). IC, now considered as a major pest in India, causes up to 20% yield loss in jute cultivation (Ramasubramanian et al., 2009). IC damages the shoots of seedling near the bottom. This causes significant reduction in the plant stand. *C*. *capsularis* is more susceptible to IC infestation than *C. olitorius* when grown early in April (JAF EXPERT, 2017).

Management of insect infestation requires repeated application of insecticides on jute crop. The relative resistance of HC larvae and their dense body hair reduce insecticidal action (Dhingra et al., 2007). IC larvae show resistance against a wide range of insecticides such as organophosphates, carbamates and pyrethroids (Wolfenbargaer, 2002). Besides increasing the cultivation cost, repeated applications of chemical insecticides negatively affect farmer’s health, other beneficial insects, soil microbes and herbivores. Alternatively, for sustainable pest management bio-pesticides are available in the market, of which 2% are based on entomopathogenic gram-positive soil bacteria *Bacillus thuringiensis* Berliner (Bt) (Bravo et al., 2011). Bt spores containing crystal proteins (Cry) known, as δ-endotoxins are insecticidal in nature. The ingested Bt toxin affects specific insects by binding to their cilial brush border receptors thereby opening membrane pores of the gut epithelium. This disrupts the transport of solutes, causing an influx of water followed by cell swelling and lysis. The highly specific mode of action of Bt, requiring specific receptors, proteases and an alkaline pH, renders it harmless to mammals (that have an acidic gut and lack the corresponding receptors).

Bt formulated bio-insecticides are also effective against the major jute pests like SL (Das and Singh, 1976), HC (Bandyopadhyay et al., 2014) and IC (Zhu et al., 2006). The requirement for repeated bio-insecticide application, due to chances of being washed out by rain, dewdrops and wind along with inactivation of toxin due to heat, sunlight and UV light exposure, increases the overall cultivation cost (Sanahuja et al., 2011). These limitations of Bt formulations can be overcome through creation of genetically modified (GM) crop expressing the key insecticidal component, the Bt crystal endotoxin(s) for resistance to insect pests.

Among the different types of *cry* genes identified, the *cry1Ab* and *cry1Ac* genes have proved successful in most commercial events of insect resistant (IR) transgenic plant development (ISAAA, 2017). The remarkable adaptability of insects to insecticides is a persisting concern for Bt crop’s success. Recently some cases of insect adaptation were reported- maize stalk borer (*Busseola fusca*) to *Cry1Ab* in Bt corn in South Africa, corn earworm (*Helicoverpa zea*) to *Cry1Ac* in Bt cotton in United States, pink bollworm (*Pectinophora gossypiella*) to *Cry1Ac* in Bt cotton in India and cotton bollworm (*Helicoverpa armigera*) to *Cry1Ac* in Bt cotton in northern China (Tabashnik et al., 2013; An et al., 2015). To delay this insect adaptation approaches like “second generation” Bt crops, with 2 or more combined Bt toxins with different modes of action and high level of expression (Carriere et al., 2015) or “High Dose/Refuge Strategy” (HD/R) (Gryspeirt and Gregoire, 2012) was practiced in Bt crop cultivation with considerable success confirmed by the 15 years HD/R resistance management strategy for Bt crops in North America (Huang et al., 2011). The fusion of *cry1Ab* and *cry1Ac* genes (*cry1Ab/Ac*) under rice *actin1* constitutive promoter was successfully reported in rice plants against yellow stem borer (*Scirpophaga incertulas*) (Tu et al., 1998), striped rice stemborer (*Chilo suppressalis*), pink stem borer (*Sesamia inferens*), leaf folder (*Cnaphalocrocis medinalis*) and green semilooper (*Naranga aenescens*) (Tu et al., 2000; Ye et al., 2001) and in chickpea plants against pod borer (*Helicoverpa armigera*) (Ganguly et al., 2014).

To curb the immense jute yield losses and retain its fiber quality by keeping away from infestation of *Lepidopteran* insect pests in India, we applied the genetic breeding approach for development of IR jute. The IR transgenic jute lines constitutively expressed the fused Cry1Ab/Ac endotoxin and the mortality of *Lepidopteran* insect pests was confirmed by analysis of all the transgenic progenies. In this investigation, our objectives were (i) to develop a T-DNA construct of fused cry1Ab/Ac genes driven by rice actin1 promoter and to modify the jute genome with it by *Agrobacterium* mediated shoot tip transformation; (ii) to select and validate the best transgenic lines; (iii) to measure the level of resistance against 3 major *Lepidopteran* pests of jute- SL, HC and IC by detached leaf and whole plant bioassay; (iv) to evaluate the agronomic and fiber quality parameters of different transgenic lines.

## Materials and Methods

### Plant Material and Explant (Shoot Tip) Preparation

**FIGURE 1:**
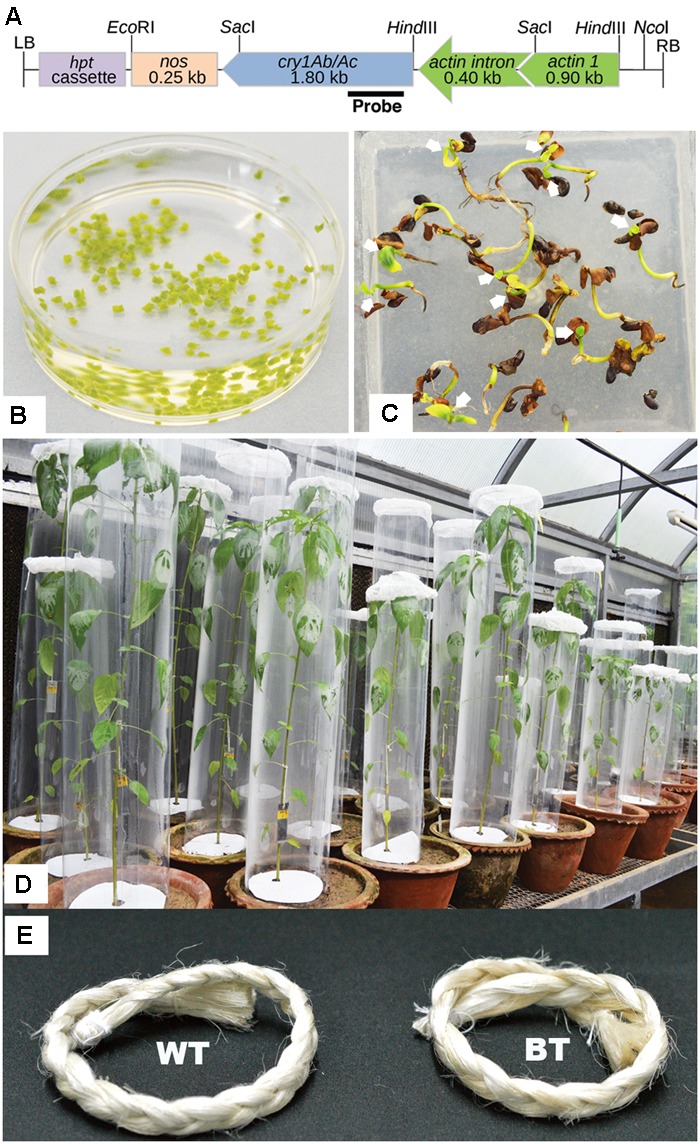
Stages of Bt jute development. **(A)** Schematic map of transformation vector pCAMBIA1301-*actin-cry1Ab/Ac-nos* (without *gus*) containing rice actin constitutive promoter (with its first intron), fused *cry1Ab/Ac* gene and nos terminator. Marked site for *Nco*I and portion in the gene was used as probe for Southern analysis **(B)** Isolated shoot tips from 5 day-old plants in infiltration media containing LBA4404 *Agrobacterium* cells. **(C)** Transformants (arrow marked) selected in hygromycin-B containing MS media remain healthy whereas non-transformed seedlings show browning and die. **(D)** Each transgenic plant of 90 DAS age, was subjected to WPB in the greenhouse with 10, second instar larvae. **(E)** Fibers of Bt jute and WT (non-transgenic) were compared after extraction where no significant differences were found in color, texture and quality.

Seeds of white jute (*C. capsularis*) of variety JRC 321 were obtained from ICAR-CRIJAF, Barrackpore, Kolkata, India (Latitude: 22°45.28’N, Longitude: 88°25.32′N). Seeds were surface sterilized by immersing in 70% ethanol for 5 min, followed by shaking in a mixture of sodium hypochlorite with Tween 20^®^ for 20 min and lastly given 5 consecutive rinses in sterile water. Then the seeds were soaked on filter paper and were grown on “germination media” (**Table 1**) in culture room (28°C, 16 h light and 8 h dark photoperiod). Shoot tips were dissected out from 5 to 10-day-old seedlings for transformation in sterile condition (**Figure 1B**).

**Table 1:** Composition of different culture media used for Bt jute development.

Plant media name	Composition	pH
Germination media	MS salts and vitamins (HiMedia Lab. Pvt. Ltd., Mumbai, India) + Sucrose (15%) + Agar (0.8%)	5.8
Infiltration/Co-cultivation media	MS salts and vitamins + Myo-inositol (0.1%) + Sucrose (2%) + BAP (0.5 mg/l) + IAA (1 mg/l) + GA_3_ (0.2 mg/l) + Acetosyringone (20 mg/l)	5.6
Agro-eradication media	MS salts and vitamins + Sucrose (15%) + Timentin (500 mg/l)	5.6
Elongation/Selection media	MS salts and vitamins + Myo-inositol (0.1%) + Sucrose (3%) + BAP (1 mg/l) + IAA (0.5 mg/l) + Hygromycin-B (12 mg/l) + Agar (0.8%)	5.8
Rooting media	Half strength MS salts and vitamins + Myo-inositol (0.05%) + Sucrose (1.5%) + IBA (0.3 mg/l) + Agar (0.6%)	5.8
Segregation + Selection media	MS salts and vitamins + Sucrose (15%) + Hygromycin-B (15 mg/l) + Agar (0.8%)	5.8

*Here, BAP, N6-Benzylaminopurine; IAA, Indole-3-acetic acid; IBA, Indole-3-butyric acid; GA_3_, Gibberellic acid and MS is for Murashige and Skoog (1962).*

### Plant Transformation Vector Construction

Construction of a reporter free transformation vector required removal of a 2054 bp fragment containing *b-glucuronidase* (*gus*) gene from the pCAMBIA 1301 (accession number AF234297) by *Bst*EII–*Bgl*II digestion, klenow treatment and self-ligation. In this vector the rice *Actin1* promoter and nopaline synthase (*nos*) terminator fragments were cloned at *Hin*dIII/*Sac*I and *Sac*I/*Eco*RI, respectively. *Sac*I digested fragment, containing the 0.40 kb of first 5′ intron of rice *actin* gene with fused 1.8 kb of *cry1Ab/Ac* gene, was released from the vector pFHBT1 (Tu et al., 1998, 2003) and ligated at *Sac*I site of the pCAMBA1301 to generate *pCAMBIA1301*-*actin*-*cry1Ab/Ac*-*nos* construct (**Figure 1A**). Nucleotide sequences of promoter, *cry1Ab/Ac* gene and terminator are available in database (accession number EU880444.1). The *hygromycin phosphotransferase* (*hpt*) gene, whose product detoxifies the aminocyclitol antibiotic hygromycin-B, was used as a marker.

### Plant Transformation and Regeneration

Shoot tips were isolated from 5 to 10 days old seedlings and delicately pierced nearly the apical meristematic dome with a sterile needle for easy penetration of *Agrobacterium.* Shoot tips were then immersed in the *Agrobacterium* “infiltration/co-cultivation media” for 1 h in dark condition before vacuum infiltration. Suspension media contained about 1.488 × 10^8^ cell/ml (OD at 600 nm 0.3) *Agrobacterium* (LBA4404) cells harboring the *pCAMBIA1301*-*actin*-*cry1Ab/Ac*-*nos* construct. Isolated shoot tips were vacuum infiltrated under a pressure of 600 mm Hg for 10 min. Inoculated shoot tips were soaked in filter paper for removing excess *Agrobacterium* suspension and placed on Whatman No.1^TM^ filter paper in a 90 mm petri plate with their apices upward. Finally 3 ml of the co-cultivation media was added, sealed with PARAFILM^TM^ and kept at 28°C dark conditions for 72 h for co-cultivation. The co-cultivated shoot tips were washed 3–4 times in “Agro-eradication media” (**Table 1**). After blotting dry on sterile filter paper, shoot tips were transferred to the same timentin antibiotic supplemented media and grown in culture room condition for a week. This week is called the “recovery period” to kill of any residual *Agrobacterium* and a time given for shoot tips to recover from physical stress applied during vacuum infiltration. Then shoot tips were transferred to shoot tip “elongation/selection media” (**Table 1**). Transformed shoot tips were selected by 3 consecutives selections at fortnightly intervals on 12 mg/l hygromycin-B supplemented media. Selected shoot-tips were transferred to “rooting media” (**Table 1**) without any selection pressure for rooting. About 8 weeks after transformation, surviving healthy plants were transferred to a greenhouse in soil supplemented with N:P:K::60:30:30.

### Germination and Mendelian 3:1 Segregation Test in Hygromycin-B Containing Media

Mendelian segregation analysis of transgene was performed by germinating transgenic seeds of T_1_ and T_2_ progenies in 15 mg/l hygromycin-B containing “segregation + selection media” for duration of 30 days (**Table 1**). Selection media was replaced after 15 days. Survival percentage and phenotypic appearance of seedlings were recorded in comparison to non-transgenic plants.

### Chlorophenol Red (CPR) Assay for Qualitative Detection of *hpt* Gene

CPR assay media was prepared by adding filter sterilized 1000 mg/l (w/v) Chlorophenol red (Sigma–Aldrich, St. Louis, MO, United States) and 15 mg/l hygromycin-B in liquid half-strength MS (salts and vitamins with 15% (w/v) sucrose, pH 5.6). Surface sterilized leaves (25 mg) were submerged with 5 ml CPR assay media in a 35 mm petri plate, sealed with PARAFILM^TM^, kept in culture room conditions for 72 h (Wright et al., 1996). Finally leaf samples were scored as positive (yellow and orange color) or negative (red and purple color) according to change in color observed and media pH was measured. For each plant 3 biological replications were taken and pH was recorded by 5 reads by the pH meter.

### Genomic DNA Extraction and PCR Screening

Genomic DNA was isolated from young leaves using NUCLEOSPIN PLANT II (Macherey-Nagel, Neumann Neander Str. Germany). All T_0_ events as well as their progeny plants were screened for *cry1Ab/Ac* and *hpt* genes by polymerase chain reaction (PCR) with 100 ng genomic DNA. PCR primers for *cry1Ab/Ac* (BTF-5′-CGGATCCGATCTTCACCTCAGCGTGCTT-3′ and BTR- 5′-CGAGCTCGGGCACATTGTTCTGTGG-3′) and *hpt* (HPTF 5′-CGCCGATGGTTTCTACAAAGA-3′ and HPTR 5′-TCAATGACCGCTGTTATGCG-3′) were synthesized through Integrated DNA Technologies, United States. The annealing temperature was 55.0°C for *cry1Ab/Ac* and 58.4°C for *hpt*. The amplified products were subjected to electrophoresis on 1% agarose gel with 1 kb Generuler ladder (Thermo Scientific, Waltham, MA, United States).

### Southern Blot Analysis

Southern hybridization with T_4_ plants was performed according to a standard protocol (Sambrook and Russell, 2001). Genomic DNA (15 μg) was digested with *Nco*I, separated on 1% agarose gel and transferred to a positively charged nylon membrane. As per manufacturer’s instruction (Roche, Basel, Switzerland), a probe (746 bp PCR amplified product of *cry1Ab/Ac*) was labeled with digoxigenin (DIG)-dUTP and detected by enzyme immunoassay through DIG DNA labeling and detection kit.

### Total RNA Isolation, cDNA Synthesis, Reverse Transcriptase (RT) PCR Analysis and Quantitative Real-Time PCR (qRT-PCR)

Total RNA was isolated from 100 mg fully opened young leaf from transgenic and non-transgenic control plants by using NUCLEOSPIN RNA PLANT isolation kit (Macherey-Nagel, Neumann Neander Str. Germany). For RNA, RT and qRT-PCR analysis advanced generation plants of T_3_ and T_4_ progenies of 40–120 days after sowing (DAS) were used. The purified RNA was treated with DNase (Thermo Fisher, Waltham, MA, United States) to eliminate genomic DNA contamination. The cDNA was synthesized using 1 μg of total RNA in iSCRIPT RT SUPERMIX cDNA synthesis kit (BioRad, Hercules, CA, United States) following the manufacturer’s instructions. The qRT-PCR reaction was performed in CFX 96^TM^ Real time system (Bio-Rad, Hercules, CA, United States), in triplicates. Jute *26S ribosomal RNA* gene (Accession number JK743816) was used as endogenous control to normalize all data. The qRT-PCR reaction mixture comprised of *cry1Ab/Ac* specific primers (RTF5′-GACTGCTGGAGTGATTATCGACAGA-3′, RT5′-AGCTCGGTACCTCGACTTATTC AG-3′), *26SrRNA* primers (F5′-GTTCCACACGAGATTTCTGTTC-3′, R5′-TTTTAGACCCAAGACC GGC-3′) and MAXIMA SYBR GREEN^®^(Thermo Scientific, Waltham, MA, United States). Quantitative variation among different samples was determined using the 2^-ΔΔC_T_^ method. All the data were analyzed using Bio-Rad CFX MANAGER^®^ software (BioRad, Hercules, CA, United States). The cDNA was also used in semi-quantitative RT PCR for amplification of *cry1Ab/Ac* gene product.

### Immunoassay for Quantitative Estimation of Bt-Protein

Sandwich enzyme linked immunosorbent assay (ELISA) was performed with T_4_ progenies of 90 DAS aged transgenic plant samples for quantitative estimation of Cry1Ab/Ac protein. All steps were followed according to manufacturer instructions (Krishen Biosystem, Ashley Ct., CA, United States) with 3 technical replications. Young opened 100 mg leaves were crushed with a pestle by adding 1 ml of extraction buffer (supplied by manufacturer) and the extract was used for the assay. Negative control, standards and sample extract were added to each well in the microtiter plate in triplicates. Absorbance at 450 nm was measured by blanking on the zero standard (blank) in a microplate reader (BioRad, Hercules, CA, United States). Concentration of Cry1Ab/Ac proteins in transgenic plants was calculated from standard curve keeping dilution factor under consideration.

### Insect Rearing

The incipient colonies of HC, SL and IC were obtained from ICAR-CRIJAF Research Farm. The F_1_ population of insects were cultured on *C. capsularis* (JRC-321) leaves in a round rearing container (0.13 × 0.13 m) with a netted lid for aeration. To avoid microbial contamination, feed and rearing containers were changed every alternative day. The F_1_ progeny of laboratory-reared larvae were used for bioassay.

### Insects Bioassay of Transgenic Bt Jute Plants

#### Detached Leaf Bioassay (DLB)

Area of transgenic leaves was measured by placing each on graph paper. After measurement the leaves were properly cleaned and transferred on moist filter paper in 140 mm petri plates. Second instar, starved larvae of average length 5.0 mm and weight 8.0 mg were used for bioassay. Each larva was placed on a young freshly opened leaf from 90 days old plant and observed for leaf area consumption by it. The experimental setup contained replicates of 5 leaves and 5 larvae, each in an individual plate as a single larva/leaf/petri dish. Bioassay was carried out in culture room for 72 h in duplicate sets. Comparisons of food intake (consumed leaf area) and dry weight of excreta by transgenic plant feeders and non-transgenic control plant feeders (from 5 leaves in each experiment) was done cumulatively.

#### Whole Plant Bioassay (WPB)

For WPB, the entire potted transgenic plants, of similar age and height, were covered with cylindrical mylar sheets (250 microns, optically clear and transparent), securing the top ends of these cylinders with cotton nets to avoid larval escape (**Figure 1D**). Aseptic conditions were maintained in the greenhouse to prevent infection and parasitization. After measuring their mean weight and length, 10 pre-starved, second instar larvae of HC, SL and IC were placed on each plant. Observation on larval mortality and growth (length and weight) was recorded after 10 days when maximum mortality was achieved. Meanwhile plants were watered regularly to maintain normal growth. Original larval mortality for individual bio-assayed plant was calculated applying the following formula, Larval Mortality % = (Number of dead larvae/Total number of larvae) × 100 which was further corrected if any mortality was observed in non-transgenic control plants due to natural death by using the Abbott’s (1925) formula^1^. Mean larval length and weight was calculated after DLB as total larval length or weight gained divided by number of survived larvae.

### Expression Study in Different Jute Tissues and Plant Developmental Stages

Quantitative estimation of Bt protein in different plant tissues (leaf, stem, seed coat and root) was done after randomly selecting 3 positive plants from each transgenic line. Expression of *cryAb/Ac* gene was consistently monitored from seedling (30 days) to harvesting stage (120 days) of a Bt-plant through qRT-PCR.

### Fiber Retting and Quality Measurement

Randomly selected 10 transgenic plants from each transgenic line (110 days old) and wild type, were biologically retted with pond water in a rectangular chamber for 15 days at 28–34°C. Prior to retting all samples were measured for full plant height, stem length and basal stem diameter. Fiber was extracted individually, sun dried and recorded for maximum length, fiber strength (g/tex), fiber fineness (tex) and color.

### Statistical Analyses

All the statistical analyses were performed using the Graph Pad Prism 6 software^2^. The experimental data, of 3 or more replicates, are presented as mean ± standard error (S.E.). The means and differences between group means were compared by ANOVA keeping statistical significance (*P* < 0.05) and Tukey’s multiple comparisons under consideration.

## Results

### Development of Transgenic Plants from Shoot Tips by *Agrobacterium*-Mediated Transformation

Shoot tips were isolated from 5 day-old seedlings (**Figure 1B**), followed by infiltration with *Agrobacterium* LBA4404 strain, harboring a *pCAMBIA1301*-*actin*-*cry1Ab/Ac*-*nos* construct (**Figure 1A**), as per previously standardized jute shoot tip transformation method with some modifications (Saha et al., 2014a). Transgenic plants (T_0_) were developed, selected by hygromycin-B (12 mg/l), transferred to soil and grown in greenhouse. A total of 10 T_0_ transgenic Bt jute lines were developed with the mean transformation efficiency of 4.00%.

### The *cry1Ab/Ac* Gene Integration Confirmed by PCR, Southern Hybridization, RT-PCR and qRT-PCR Analysis

**FIGURE 2:**
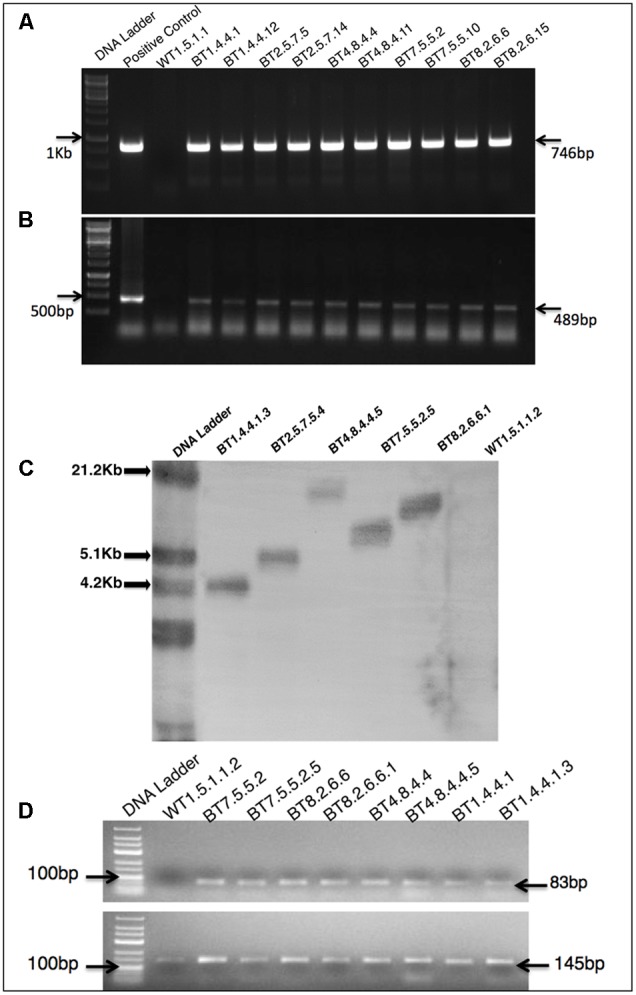
Molecular biology characterizations of transgenic plants. PCR analysis of T_3_ transformants, showing partial amplification of **(A)** 746 bp *cry1Ab/Ac* gene **(B)** 489 bp *hpt* gene. **(C)** Southern blot analysis of genomic DNA of T_4_ plants digested with *Nco*I and probed with DIG labeled 746 bp of *cry1Ab/Ac* showing single copy integration. **(D)** RT-PCR analysis of T_3_ and T_4_ plants of a same transgenic line showing 83 bp amplification of *cry1Ab/Ac* gene in transgenic plants and 145 bp *26S rRNA* gene (internal control) in all plants.

PCR analysis of the T_0_ transformants genome confirmed the presence of 746 bp *cry1Ab/Ac* (**Figure 2A**) and 489 bp *hpt* genes (**Figure 2B**). This process was repeated for every subsequent generation. Southern hybridization was performed with T_4_ transgenic progenies with *cry1Ab/Ac* gene specific digoxigenin-dUTP labeled probe. Single copy integration of *cry1Ab/Ac* gene at different locations of the genome was found in progenies of BT1, BT2, BT4, BT7 and BT8 transgenic jute lines (**Figure 2C**).

**FIGURE 3:**
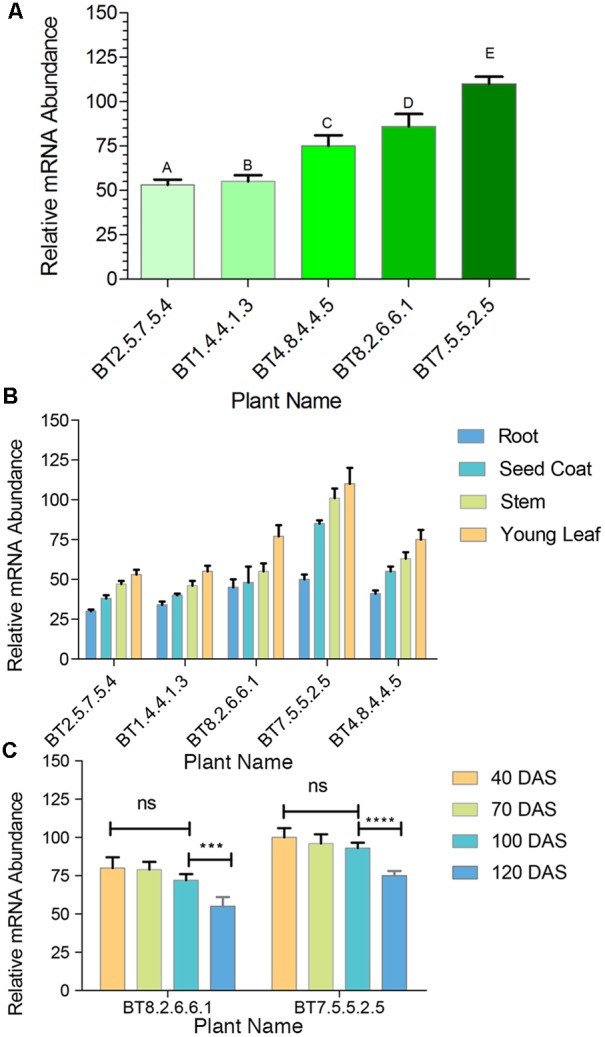
qRT-PCR comparative analysis of mRNA transcript of transgenic plants. **(A)** Bar diagram showing significant difference among transgenic lines A–C, A–D, A–E, B–C, B–D, B–E, C–E and D–E = P < 0.0001, C–D = P < 0.0014 in the Tukey’s multiple comparisons test where A-B found to be non-significant. The BT8.2.6.6.1 and BT7.5.5.2.5 lines show highest expression. **(B)** qRT-PCR analysis showing highest expression of mRNA transcript in young leaves compared to stem, seed coat and root. **(C)** Non significant (ns) changes in *cry1Ab/Ac* gene expression between 40 and 100 DAS was observed but from 100 to 120 DAS a significant change was found in BT7.5.5.2.5 (^∗∗∗∗^*P* < 0.0001) and BT8.2.6.6.1 (^∗∗∗^*P* < 0.0015) plants. Each bar represents the mean ± standard error (SE) of 3 independent experiments. Here *cry1Ab/Ac* gene expression was calculated on the basis of internal control *26S rRNA* gene expression.

Comparative study of gene expression among different transgenic generations of each transgenic line was conducted through RT-PCR and qRT-PCR. In RT-PCR, transgenic plants revealed 83 bp of *cry1Ab/Ac* transgene along with 145 bp of *26sRNA* gene (internal control). Similar expression (band intensity) manifested in the T_3_ and T_4_ progenies of every transgenic line (**Figure 2D**). Similar kind of result was found in qRT-PCR analysis of T_2_ and T_3_ progenies (Supplementary Figure S1). The *cry1Ab/Ac* expression level in the BT7 transgenic line was 110 fold higher than the level of *26SrRNA* expression, whereas the wild type plant did not show any expression (**Figure 3A**).

Expression was highest in young leaves followed by stem, seed coat and root, with significant difference (**Figure 3B**). This analysis was done at regular intervals during plant growth, starting from 40 DAS upto fiber harvesting time (120 DAS). A significant drop in *cry1Ab/Ac* expression level was observed after 100 DAS for BT7.5.5.2.5 and BT8.2.6.6.1, the 2 most expressive T_4_ progenies (**Figure 3C**).

### The *hpt* Gene Inheritance Analysis in Hygromycin-B Containing Media and CPR Assay

The transformed jute contained *hpt* marker gene, whose product detoxifies the aminocyclitol antibiotic hygromycin-B. Inheritance of *hpt* gene in T_1_ and T_2_ progenies was confirmed by survivable percentage of plants in hygromycin-B (15 mg/l) supplemented media. In the presence of hygromycin-B, nearly all seeds germinated initially but after 5-7 days distinct phenotypic changes were observed in some seedlings, showing browning of stem and root, rapid degradation of cotyledon chlorophyll, stunted growth, no lateral root formation and necrosis, thereby confirming *hpt* gene inheritance by only the healthy seedlings (**Figure 1C**). The Mendelian principle of 3:1 segregation of *hpt* gene was analyzed from the survivable percentage (**Table 2**).

**Table 2:** Segregation analysis of *hpt* gene in T1 and T2 generation progeny plants.

Transgenic generation	Plant ID	*hpt* positive	*hpt* negative	Plant survivable % in hygromycin-B	Best fit segregation ratio	Chi squared value	*P*-value
T_1_	BT1	34	10	77.27	1:3	0.12	0.73
	BT2	40	9	81.63	1:3	1.15	0.28
	BT4	35	13	72.92	1:3	0.11	0.74
	BT7	34	15	69.39	1:3	0.82	0.36
	BT8	35	12	74.47	1:3	0.07	0.93
							
T_2_	BT1	41	12	77.35	1:3	0.16	0.70
	BT2	44	21	67.69	1:3	1.85	0.17
	BT4	50	15	77.00	1:3	0.13	0.72
	BT7	49	22	69.01	1:3	1.36	0.24
	BT8	46	20	69.70	1:3	1.00	0.31

**FIGURE 4:**
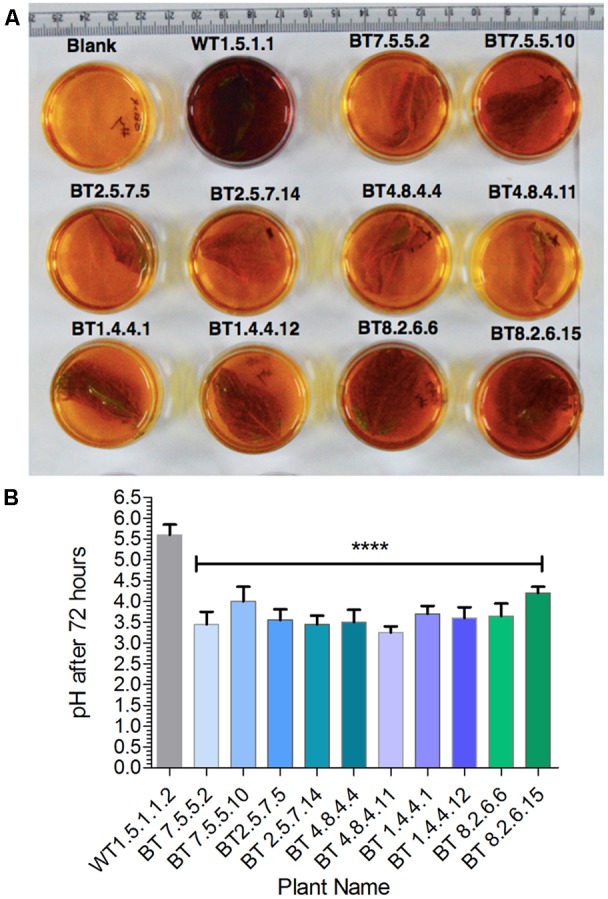
Screening of transgenics by CPR assay. **(A)** After incubation in hygromycin-B, transgenic plants developed yellow color with significant difference (^∗∗∗∗^*P* < 0.0001) in media pH value as compared to non-transgenic (WT). **(B)** Bt jute leaf containing media showing pH range of 3.0–4.12.

Plants of T_3_ and T_4_ generation, grown under greenhouse conditions, were subjected to confirmation test for the presence of *hpt* gene by pH indication property of CPR assay (**Figure 4A**). After 72 h incubation period color of the media changed from yellow to dark reddish-purple with pH > 4.5 for the negative plants and the color remained almost unchanged for positive plants with the mean pH range 3.0–4.12 (**Figure 4B**). Significant difference of mean (*P* < 0.0001) was found in the pH level of *hpt* positive, negative and WT plants.

### Quantification of Cry1Ab/Ac Endotoxin in Transgenic Progenies by ELISA

**FIGURE 5:**
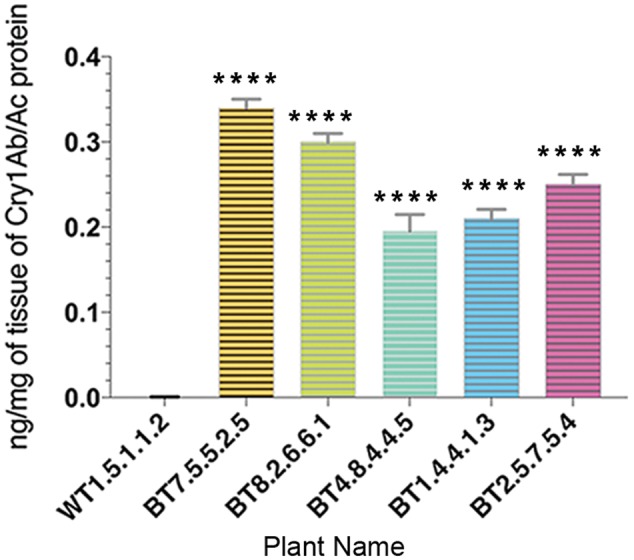
Enzyme linked immunosorbent assay quantification showing variation in Cry1Ab/Ac protein expression in different transgenic plant lines. All data were expressed as the mean ± SE of 3 replicates. Significant difference (^∗∗∗∗^*P* < 0.0001) in endotoxin expression in transgenics compared to non-transgenic (WT) in T_4_ generations.

The quantitative estimation of Cry1Ab/Ac by sandwich ELISA provided results of significant difference in expression levels of Cry1Ab/Ac endotoxin among leaf protein samples from transgenic lines of 90 DAS. Cry1Ab/Ac protein was in the range of 0.16–0.35 ng/mg in the samples where the highest expression was seen in progenies of the BT7 line (**Figure 5**).

### Effect of Cry1Ab/Ac Endotoxin on HC, SL and IC Larvae

**FIGURE 6:**
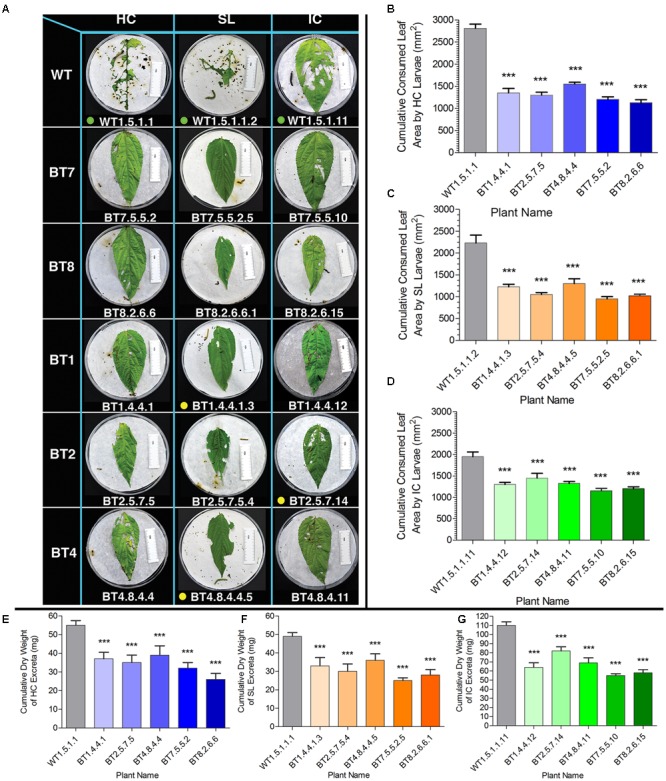
Comparative analyses of leaf area consumption and dry weight of excreta after detached leaf bioassay (DLB) by SL, HC and IC larvae. **(A)** Representative photograph of HC, SL and IC after 96 h of feeding (when maximum mortality was observed). Larvae consumed more leaf area of WT than transgenic plants. Here green dots signify alive larvae, yellow dots for paralyzed and no dots for dead larvae. The length of the white ruler in the picture is of 50 mm and size of petri plate is 140 mm. Arrows in WT set of HC indicate parts of head molting during larval development. **(B–D)** Cumulative leaf area consumption by 5 larvae for each plant. **(E–G)** Cumulative difference in dry weight of larval excreta after feeding on WT and Bt jute in DLB. SE was calculated from 2 biological replicates. Significant difference (*P* < 0.001) denoted as ^∗∗∗^ in transgenics compared to WT.

Cumulative consumption of food (leaf area) in WT feeders in DLB was 2812.00 mm^2^ for HC, 2230.00 mm^2^ for SL and 1950.00 mm^2^ for IC and that of Bt jute feeders was lower than 1550.00 mm^2^ for HC, 1300.00 mm^2^ for SL and 1450.00 mm^2^ for IC (**Figures 6A–C**). Significant difference (*P* < 0.01) was estimated for HC, SL and IC larvae feeding on WT and transgenic jute. IR ability based on consumed leaf area of the BT7 and BT8 line plant progenies were found to be superior than BT1, BT2 and BT4 line plant progenies against the larvae of the 3 test insects found in DLB (**Figure 6A**).

**FIGURE 7:**
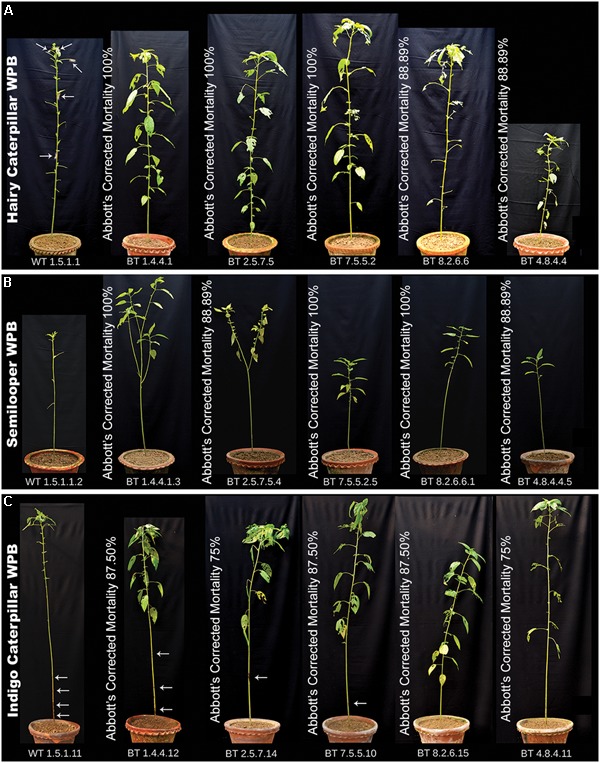
Bt plants showing resistance to HC, SL and IC infestation in WPB. Each plant bio-assayed with 10 second instar larvae. Photograph for comparison of Bt jute and non-transgenic plants (WT) was taken on final day of bioassay and represent with the Abbott’s corrected larval mortality percentage. All WT plants showing complete damage by insects whereas transgenic lines showing insect resistance. **(A)** Bioassay with HC. Arrows showing some living insect in WT plant. **(B)** Bioassay with SL and **(C)** Bioassay with IC. Arrows showing location of damage in stem by IC in WT and Bt jute plants.

In WPB the Abbott’s (1925) corrected larval mortality percentage recorded on 10 DAF (days after feeding) was 88.89–100% for HC and SL and 75–87.50% for IC, feeding on progeny plants of transgenic lines (**Figure 7**). Mean of corrected larval mortality percentage for 5 most potent lines (BT1, BT2, BT4, BT7, and BT8) has been represented in **Table 3** where mortality of larvae feeding on all bio-assayed PCR positive Bt jute plants has been taken under consideration.

**Table 3:** Transgenic Bt jute lines and their mortality (%) in WPB.

Bt jute Lines	HC	SL	IC
BT1	92.00 ± 2.23 #AP = 10	97.00 ± 1.82 #AP = 10	70.00 ± 2.97 #AP = 10
BT2	98.75 ± 2.72 #AP = 10	96.25 ± 1.27 #AP = 9	75.50 ± 2.65 #AP = 10
BT4	97.44 ± 2.29 #AP = 9	95.55 ± 1.29 #AP = 9	62.22 ± 2.88 #AP = 9
BT7	98.66 ± 2.23 #AP = 10	99.00 ± 1.77 #AP = 10	87.50 ± 2.08 #AP = 7
BT8	96.66 ± 1.66 #AP = 9	99.11 ± 1.82 #AP = 10	85.66 ± 3.16 #AP = 8

*Here #AP is number of assayed plants (PCR positive advanced Bt jute plant progenies) of the particular transgenic line. Values are presented as mean of Abbott’s corrected larval mortality % ± standard error (*n* = #AP).*

**FIGURE 8:**
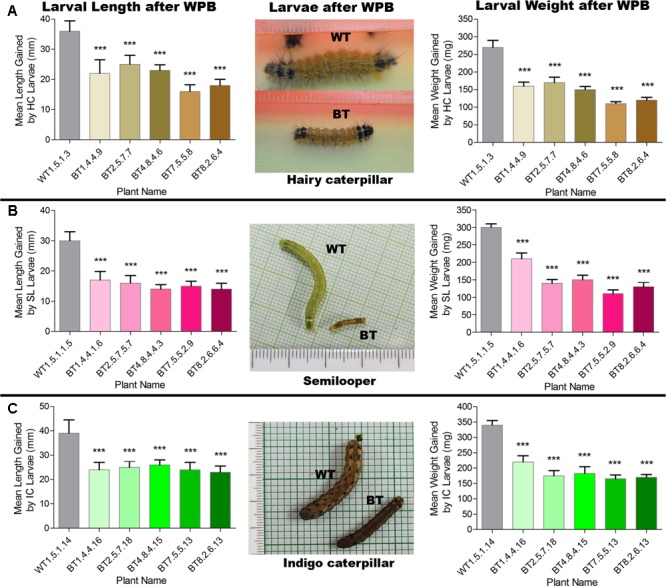
Comparative analyses of mean length and weight gained by larvae **(A)** HC, **(B)** SL and **(C)** IC after whole plant bioassay (WPB) on host Bt jute (BT) and non-transgenic (WT) plants. Significant difference (*P* < 0.001) denoted as ^∗∗∗^ in Bt jute compared to WT jute.

Most of the transgenic feeding larvae were dead after 7–9 DAF and the rest were paralyzed. The mean gain in length and weight was compared between transgenic and non-transgenic feeders after WPB (**Figure 8**). Transgenic feeding larvae achieved mean length of 25.00 mm for HC, 17.00 mm for SL and 26.00 mm for IC whereas WT feeding larvae grew up to 36.00 mm for HC, 30.00 mm for SL and 39.00 mm for IC (**Figure 8**). In case of mean weight gained after WPB it was found that transgenic feeding larvae achieved mean weight 170.00 mg for HC, 210.00 mg for SL and 220.00 mg for IC whereas WT feeding larvae weighed up to 270.00 mg for HC, 300.00 mg for SL and 340.00 mg for IC (**Figure 8**). Difference in weight and length gained by larvae fed on Bt transgenic and WT jute plants showed significantly less growth (*P* < 0.0001) in Bt jute feeders compared the larvae on WT plants. Transgenic plants suffered negligible damage of foliage and stem compared to WT plants where apart from immense foliage damage, IC devoured stems from 5 to 10 cm above the soil (**Figure 7C**). During the WPB, the 3 insects SL, HC and IC that fed on transgenic plants, could not complete their life cycle due to premature death of larvae, but the ones fed on control WT plants successfully completed the developmental stages, when further maintained after bioassay. Body color of transgenic fed HC and SL larvae turned to brown then black followed by mortality.

### Agronomical and Fiber Quality Comparative Analysis among Transgenic and WT Plants

The agronomical and fiber quality variables like plant height, stem length, basal stem diameter, fiber strength and fineness between transgenic and WT plants were non significant and at par (**Table 4**). Appearance and color of the fiber was found to be same among them (**Figure 1E**).

**Table 4:** Comparative analysis of fiber quality and other agronomic characters among non-transgenic (WT) and transgenic (BT) T_4_ progenies in greenhouse condition.

Agronomic trait	Non-transgenic jute (WT)	Transgenic Bt jute lines				
		BT1	BT2	BT4	BT7	BT8
Plant height (m)	3.48 ± 0.12	3.30 ± 0.30	3.49 ± 0.21	3.60 ± 0.20	3.45 ± 0.17	3.50 ± 0.23
Stem length (m)	3.10 ± 0.17	2.95 ± 0.15	3.19 ± 0.16	3.25 ± 0.26	3.19 ± 0.21	3.17 ± 0.20
Basal stem diameter (mm)	20.0 ± 0.90	21.2 ± 1.1	19.1 ± 1.55	20.1 ± 1.23	20.3 ± 1.0	20.1 ± 0.75
Fiber length (m)	3.00 ± 0.30	3.00 ± 0.41	3.20 ± 0.22	3.22 ± 0.18	3.20 ± 0.32	3.15 ± 0.31
Fiber strength (g/tex)	19.5 ± 2.11	20.6 ± 1.27	19.6 ± 2.81	21.2 ± 1.67	22.1 ± 2.09	20.7 ± 1.70
Fiber fineness (tex)	1.51 ± 0.006	1.48 ± 0.002	1.50 ± 0.001	1.49 ± 0.007	1.50 ± 0.02	1.45 ± 0.06

*The values represent the mean of 10 progeny plants from each line ± standard error.*

## Discussion

Jute, the ‘golden fiber’-producing crop, is of high economic value due to its various domestic and industrial applications worldwide. Sexual incompatibility and chromosome morphology differences may limit the improvement of jute variety through conventional breeding (Swaminathan et al., 1961; Saha et al., 2014b). Jute cultivation suffers 31–34% loss in fiber yield due to pest infestation by more than 40 species of insects, mites and nematodes (Rahman and Khan, 2006). Our research work portrays the development of IR transgenic plants by inserting the fused *cry1Ab/Ac* gene in the *C. capsularis* genome by *Agrobacterium tumefaciens* mediated gene transfer. The effect of this fused gene product on the 3 major *Lepidopteran* pests (HC, SL and IC) was studied for 4 consecutive transgenic generations. The Cry1Ab/Ac expressing transgenic plants were selected based on insect bioassay responses on the 3 major jute pests.

Transgenic plants were bio-assayed by both non-destructive (DLB) and destructive methods (WPB) against all 3-test insects (Selvi and Sakthi, 2015). Before selecting transgenic plants for WPB, insect resistance ability was initially checked against the test insects in DLB. Due to the voracious feeding nature of the 3-test insects, a single larva was used on each leaf in DLB with biological replicates. Plant progenies of 5 transgenic Bt jute lines with nominal leaf area consumption by test larvae in DLB were subjected to WPB. In DLB, larval mortality was witnessed after 48 h of feeding on transgenic jute leaves but in this study insect mortality % was calculated in WPB. We preferred WPB for insect mortality analysis over DLB for the following reasons: experimental setup is closer to natural condition, whole plants were exposed to larvae, large number of test insects could be applied on each plant (10 larvae/plant), unlike DLB, WPB did not face issues like over drying of detached leaf sample or degradation of leaf Cry proteins during bioassay period. In this study we analyzed insect mortality percentage in 2 ways, original larval mortality percentage and Abbott’s (1925) corrected larval mortality percentage for individual plant. During the 10 days WPB period HC and SL non-transgenic feeding larvae showed up to 10% mortality whereas IC showed up to 20% in some biological replicates. This unexpected mortality in control plants is considered as natural death of test larvae and it has been considered during the final mortality data collection by applying Abbott’s formula.

Bioassay revealed a common feeding pattern where initial food consumption was high with rapid increase in body weight and length in case of all 3 types of larvae (first 1–5 Days in WPB). After 48 h in DLB and 5–7 days in WPB, majority of larvae stopped feeding along with low appetite, lethargy, paralysis and flaccidity, which resulted adversely on weight and length gain and increased mortality percentage of Bt jute feeding larvae. Maximum larval mortality was recorded for WPB after 8 days with negligible gain in their body weight and length. Similar feeding and larval conditions have been reported in case of Bt tobacco expressing *cry2Aa2* operon resulting in 100% IC larval mortality (Cosa et al., 2001). After bioassay comparison in body weight, larval length, consumed leaf area and insect mortality percentage between WT and Bt jute feeding larvae with significant difference (*P* < 0.05) it was found that Bt jute had adverse effect on the growth and development of these 3 types of insects, accompanied by high mortality percentage. The dry weight of larval excreta was measured and upon comparative analysis it is concluded that it was directly proportional to consumption of leaf tissue (**Figure 6**).

Bioassay study with 3 different insects feeding on Bt jute confirmed that mortality of IC was different from that of HC and SL. IC mortality against Cry1Ac in Bt soybean has been recorded as 75.60–94.60% (Yu et al., 2013). In our study, Abbott’s corrected mortality of IC (66.66–87.50%) was lower compared to HC (100%) and SL (100%) in Bt jute in DLB (**Figure 7**). This level of mortality of IC, caused by the Cry1Ab/Ac endotoxin, is promising to combat the damage caused by this pest in jute cultivation.

Expression level of *cry1Ab/Ac* gene, analyzed in insect susceptible parts of Bt jute, was highest in leaves and then in stems (**Figure 3B**). Being mainly foliage feeders, HC and SL caused serious damage particularly to the topical part of the plants whereas IC additionally damaged the stems (**Figure 7C**). Similar expression of *cry1Ab/Ac* in rice has been recently reported as in the order leaf > stem > root (Jiang et al., 2016; Wang et al., 2016). Exact reason for this change in cry1Ab/Ac expression is yet to be identified but this is not a characteristic of the used promoter. We used plant actin promoters (from rice) likely to be active in all plant tissues because actin is a fundamental component of the plant cell cytoskeleton. The rice actin1 promoter in combination of its first 5′ intron increased gene expression that is 40-fold more than a CaMV35S constitutive promoter (McElroy et al., 1990). Such intron combined actin1 promoter has been use for Bt jute development. Highest expression of Cry protein in the jute leaf holds promise for insect mortality as the primary site of feeding by all the test insects is on leaves. Jute suffers insect infestation throughout its cultivation time. IC attacks white jute early in April, SL and HC infest in the rainy months of June to September (Gotyal et al., 2014; Selvaraj et al., 2015). Since, a continuous protection from pests throughout the growing period is required, expression level of the insect toxin Cry1Ab/Ac was analyzed from seedling to fiber harvesting stage (120 DAS). A non-significant change in *cry1Ab/Ac* gene expression between 40 and 100 DAS was observed but from 100 to 120 DAS a significant decline was found in BT8.2.6.6.1 and BT7.5.5.2.5 transgenic plants (**Figure 3C**). Similar decline in levels of Bt toxin during maturity and senescence has been reported in cotton (Poongothai et al., 2010; Shera and Arora, 2016). The amount of expressed Cry1Ab/Ac protein ranged from 0.16 ng/mg to a highest of 0.35 ng/mg of leaf. Similar range of expression has been reported in cotton with *cry1la12* under *CaMV35S* promoter showing 40% morality and growth reduction in fall armyworm (*Spodoptera frugiperda*) and cotton boll weevil (*Anthonomus grandis*) whereas *cry1Ac* + *cry2A* showed 60–100% mortality against *Heliothis* larvae (Puspito et al., 2015; Oliveira et al., 2016). In some samples, detection of Cry1Ab/Ac protein by ELISA was hinderer due to the high mucilage content in jute plants (Yamazaki et al., 2009; Kundu et al., 2011). Therefore expression studies in different tissues were conducted using qRT-PCR considering linear correlation between mRNA transcript and protein expression.

Farmers in many countries including India are reluctant in planting 20% refugee crops along with Bt crops in the field thereby causing chances of insect adaptation (Stone, 2004; Tabashnik, 2015). This refuge crop plantation can be lowered to 10% if improved Bt plants (stacked Bt protein, high protein expression, high insect mortality) are used for cultivation (Roush, 1998). Since most of the *Lepidopteran* pests of jute are polyphagous, such as HC that attacks nearly 126 plant species including jute (Singh and Varatharajan, 1999), only a minimum area under refuge crop may serve the purpose of resisting insect adaptation to Bt jute. As feeding and breeding on alternate non-transgenic parallel hosts will maintain the susceptible progeny thereby deterring the selection pressure for development of adaptation (Li et al., 2017). Till date there are no reports on pest adaptation for this synthetic fusion Cry1Ab/Ac protein. As mentioned in introduction, Bt δ-endotoxins affects only those insects that have receptors in their cilial brush border. Based on this, a long persisting hypothesis is that Bt toxin could harm non-target insects too. But with the continuous publication of reports that state otherwise for non-target field insects, this hypothesis is gradually becoming non-significant. Reports on Cry1Ab/Ac state to have no significant effect on non-target organisms like silkworm (Yao et al., 2006), planthopper, leafhopper (Chen et al., 2006) or common arthropod predators (Xu et al., 2011). Food and feed assessment studies done on monkeys (Mao et al., 2016) and mice (Xu et al., 2009) confirmed the safety of Cry1Ab/Ac protein (Koch et al., 2015). In China, Huahui1 and TT9-3/TT9-4 are 2 out of the 19 commercialized rice events containing *cry1Ab/Ac* gene (Tu et al., 2000 ; Ye et al., 2001; Chen et al., 2011; Li et al., 2016). Bt cotton is cultivated in 11.6 million hectares in India with 95% adoption and acceptance by 7.7 million farmers (Choudhary and Gaur, 2015). It reduces insecticide usage by 24%, increases yield up to 150% and leads to increased farmer income (Carpenter, 2010; Kathage and Qaim, 2012). Similar economic impact and acceptance is seen worldwide for Bt rice (Li et al., 2016), Bt corn (Edgerton et al., 2012) and Bt sugarcane (Ye et al., 2016).

This is a report on development of IR transgenic Bt jute successfully tested against 3 major *Lepidopteran* jute pests (HC, SL and IC) and the analysis of the integrated *cry1Ab/Ac* gene expression upto T_4_ generations. Upon cultivation in the field, Bt jute expressing Cry1Ab/Ac δ-endotoxin, could protect yield losses due to *Lepidopteran* pest infestation, reduce cultivation cost (minimum insecticide application), increase farmer income and show insect resistance durability with minimum refuge crop plantation. Extended analysis of Bt jute under field conditions will lead to better understanding of Bt technology as an integrated pest management system for commercial fiber crop production and its social-economic impacts.

## Author Contributions

The experiment was designed by SD and KD. The plan for execution and performance of the experiments was done by SM. Shoot-tip transformation was optimized by PS. SM, CS, PS and KD analyzed molecular biology data. SM, BG and SS analyzed insect bioassay data. SM and SS wrote the manuscript. KD and SD edited the manuscript. All authors discussed the results and commented on the manuscript.

## Conflict of Interest Statement

The authors declare that the research was conducted in the absence of any commercial or financial relationships that could be construed as a potential conflict of interest.

## Footnotes

1 http://www.ehabsoft.com/ldpline/onlinecontrol.htm#Abbott

2 graphpad.com
