# Supplementary material for: Bt Jute Expressing Fused δ-Endotoxin Cry1Ab/Ac for Resistance to Lepidopteran Pests
Source: Front Plant Sci. 2018 Jan 4;8:2188. doi: 10.3389/fpls.2017.02188 (PMC5758602; doi:10.3389/fpls.2017.02188)
Supplement: Supplementary file 1 [file Image_1.pdf]

Bt Jute Expressing Fused δ-Endotoxin Cry1Ab/Ac for Resistance to *Lepidopteran* Pests

Majumder S, Sarkar C, Saha P, Gotyal BS, Satpathy S, Datta K, Datta SK (2017) Front Plant Sci 8:2188. doi: 10.3389/fpls.2017.02188

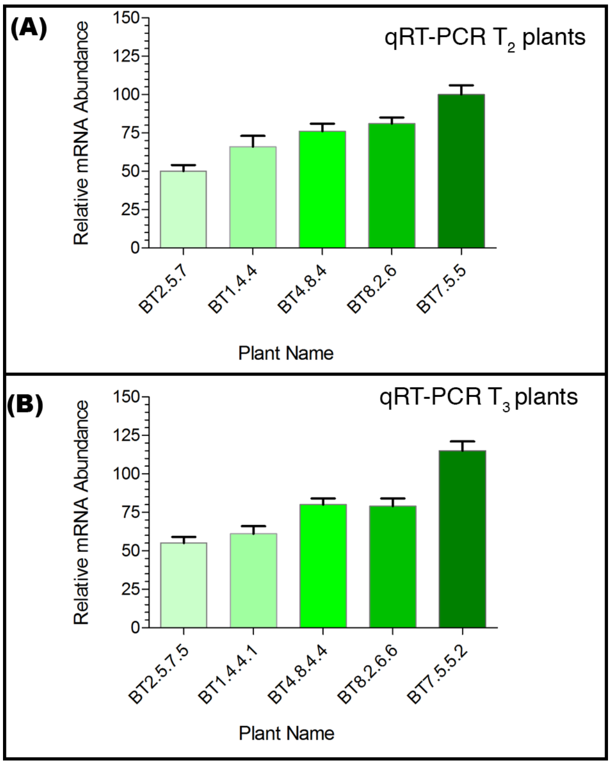

Supplementary Figure S1 qRT-PCR analysis of mRNA transcript of T_2 and T_3 transgenic plants. (A) qRT-PCR analysis of T_2 transgenic progeny plants (B) qRT-PCR analysis of T_3 transgenic progeny plants. Each bar represents the mean ± standard error (SE) of 3 independent experiments and qualified $P<0.05$ in the Tukey's multiple comparisons test. Here *cry1Ab/Ac* gene expression was calculated on the basis of internal control *26S rRNA* gene expression.
